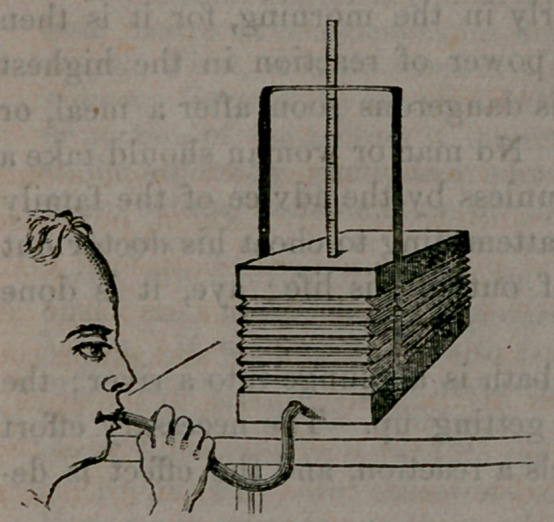# Dr. Hall’s Portable Spirometer

**Published:** 1877-12

**Authors:** 


No physician questions the value of the Spirometer for exercising and expanding the lungs, and for measuring their capacity. The great difficulty has been to find an instrument that can be compressed into a small compass when not in use, so that the physician or patient could conveniently carry it from place to place as circumstances might require. Another obstacle to the more general use of this truly valuable instrument has been its cost. The unwieldy spirometer of a few years ago, cost from $10 to $20. Dr. Hall's Portable Spirometer costs only six dollars, and will be sent free to any address on receipt of the price by the editor of this JOURNAL.

It is 6 by 12 inches, and when closed it stands only 3 inches high, and weighs less than 3 pounds.

This cut represents Dr. Hall's Portable Spirometer, partly inflated, in order to show the manner of using it. When not in use it can be closed together so as to occupy the small space above stated. The scale and guide are hinged, so that they close down on the top of the Spirometer, and occupy but little space. The brass-work is plated with silver or nickel.

HOW TO USE THE SPIROMETER.

The instrument should be used only in a perfectly ventilated room, which is entirely free from dust, or else in the open air away from the dust and smoke and bad odors of the streets of a city. When it is remembered that the capacity of the lungs of a man of medium size is from 150 to 250 cubic inches, and that in the act of breathing naturally, or rather as some have acquired the habit of breathing—which is far from natural—the lungs take in only 40 cubic inches of air at each inspiration, leaving from 110 to 210 cubic inches of air cells almost always in a dormant condition, the importance of regular and systematic exercise for the lungs will be appreciated.

It is now very generally conceded that mankind start quite evenly on the journey of life, in spite of supposed hereditary tendencies to pulmonary consumption, and that with proper care the average term of life is not shortened by these hereditary tendencies. This being the case, it is far better to enjoy health and long life through means which the naturally robust are not compelled to resort to, than to believe in or lament over the inheritance of ills that do not exist. We believe for all persons who are confined much of the time in doors, particularly in cases where there is a tendency to weak lungs, the regular use of the spirometer in the open air is highly beneficial. Its use just before bedtime promotes sleep.

## Figures and Tables

**Figure f1:**